# Design and Experimental Research of Knee Joint Prosthesis Based on Gait Acquisition Technology

**DOI:** 10.3390/biomimetics6020028

**Published:** 2021-05-07

**Authors:** Yonghong Zhang, Erliang Wang, Miao Wang, Sizhe Liu, Wenjie Ge

**Affiliations:** School of Mechanical Engineering, Northwestern Polytechnical University, Xi’an 710072, China; ErLiang_Wang@163.com (E.W.); wmjy@mail.nwpu.edu.cn (M.W.); sizheliu@mail.nwpu.edu.cn (S.L.)

**Keywords:** gait acquisition, lower limb prosthesis, instantaneous movement, bionic performance

## Abstract

Whether the lower limb prosthesis can better meet the needs of amputees, the biomimetic performance of the knee joint is particularly important. In this paper, Nokov(metric) optical 3D motion capture system was used to collect motion data of normal human lower limbs, and the motion instantaneous center of multi-gait knee joint was obtained. Taking the error of knee joint motion instantaneous center line as the objective function, a set of six-bar mechanism prosthetic knee joint was designed based on a genetic algorithm. The experimental results show that the movement trajectory of the instantaneous center of the knee joint is basically similar to that of the human knee joint, so it can help amputees complete a variety of gaits and has good biomimetic performance. Gait acquisition technology can provide important data for prosthetic designers and it will be widely used in prosthetic design and other fields.

## 1. Introduction

From a bionic perspective, a qualified prosthetic knee joint can ensure that amputation patients get a steady gait. We need to collect gait data of normal humans and analyze the gait characteristics, and then establish a knee joint prosthesis model to improve the bionic performance of knee joint prostheses.

(1) Gait analysis has attracted more and more attention in the fields of rehabilitation medicine [1], identity recognition [2], and prosthetic limb design [3]. In the field of prosthetic design, effective gait analysis can provide designers with kinematic and dynamic parameters related to amputees. At present, domestic and foreign gait information acquisition technologies mainly include image processing technology, foot sensor and wearable sensor gait acquisition technology [4]. Image processing technology is the technology that uses a computer to process the information of gait image. Foot sensors provide force analysis of the gait. Wearable sensor gait acquisition technology can automatically quantitatively analyze and evaluate the walking state and ability of subjects in actual activities, work and leisure.

With the development of camera technology, by capturing a series of images of human gait and analyzing the captured images, relevant gait parameters can be obtained. Tanawongsuwan of Georgia Institute of Technology in the United States used a motion capture system [5] to mark reflective points on human joints and then obtained corresponding gait data. This technology can obtain more accurate data, but it is expensive and difficult to move. These reasons lead to a vision system-based gait acquisition technology that can only be used in the laboratory [6].

In recent years, due to the rapid development of sensor technology, gait data collection research usually uses sensor technology [7]. Sensor technology can be divided into foot sensors and wearable sensors. The members of the Institute of Electrical and Electronics Engineers (IEEE) Stacy J. Morris Bamberg, Ari Y. Benbasat et al. have proposed a wireless sensor-based gait acquisition system called "Gait Shoe" that can be placed in shoes [8]. Li Xiufeng From the Chinese Academy of Sciences designed a data transceiver module based on a six-axis data sensor [9]. This kind of sensor design of gait analysis system with micro power consumption and low power consumption as a starting point gives full consideration to the portable gait analysis system on the system size and power requirements, and can make the acquisition node system under the condition of the normal 300 Ma lithium battery use half a month, so it has stable performance, miniature, low power consumption, low cost, and is convenient and portable etc., However, the maximum error of the experimental results is 13%, so the accuracy is not enough.

In reference [10], Chaparro-Rico et al. used the Sane gait parameter evaluation system to analyze and study the gait of 12 subjects, and verified that the system had a high reliability and great potential. The test–retest reliability statistics were obtained from 0.62 to 0.81. The wide range of interrater reliability ranged from 0.70 to 0.95 and intra-rater reliability ranged from 0.74 to 0.92, indicating that the wide range of interrater reliability ranged from 0.70 to 0.95. However, the size and sample size of the experiment need to be expanded to make SANE an application tool for evaluating gait parameters of people with lower limb disabilities. In reference [11], B.D.M. et first describes the arm movement disorders in humans, and the movement of four arm motion planning of procedures and data acquisition are described, followed by regression an analysis method to generate a reference trajectory, and its equipment is used in 12 subjects for 12 cycles of arm movement, in the track samples available. The feasibility of this method is proved.

(2) The prosthetic knee joint is the most important joint in the lower limb prosthesis. A good prosthetic knee joint must have good bionic performance, that is, the more similar the instantaneous center line of the knee joint movement is to the instantaneous center line of human knee joint, the better, so as to help the prosthetic patients achieve a variety of gaits. In people’s daily life, walking on flat ground, up and down stairs, even running and jumping, are the most basic behaviors, so the design of a prosthetic knee joint should meet these needs. In addition, existing prosthetics are being researched and designed towards these goals. The current prosthetic knee joint can be mainly divided into uniaxial and multiaxial groups in terms of structure.

The uniaxial prosthesis has only one rotation axis, and has the advantages of a simple structure, a light weight, and a simple motion relationship. Koh Inoue et al. at Kagawa University in Japan designed a single-axis knee joint prosthesis [12]. This prosthesis can enable patients to complete the movement of stairs without the help of any auxiliary equipment. The Holland Bloorview Children’s Rehabilitation Hospital in Canada also designed a single-axis knee joint prosthesis [13]. The standing phase can realize the self-locking function and the walking speed can reach 0.14 m/s. The passive uniaxial knee prosthesis designed by Arekatti [14] can realize the patient’s walking gait through the limit mechanism and springs. However, these uniaxial knee prostheses are often unable to achieve more complex trajectories, and the similarity between them and the instantaneous center trajectories of the human knee joint is low.

The knee joint movement of the uniaxial prosthesis is realized by a single hinge. Because of its simple working principle, the research on its structure focuses on the realization of special functions such as standing phase self-locking, redundant drive, up and down stairs, etc. A multi-axis prosthetic knee joint is a kind of prosthetic knee joint which is realized by a multi-bar mechanism or a gear mechanism and has multiple or unfixed rotating axes. It can realize more complex movement such as running and jumping. It has advantages in imitating and energy utilization. Altamirano a, Mexico, et al. designed a set of four link actuators for the knee joint of the prosthesis [15], and optimized the mechanism and materials to reduce costs, which can meet the needs of most patients with lower extremity amputation. The gear five bar knee prosthesis designed by Sun yuanxi of Northwest University of technology [16] can reduce the freedom of mechanism to 1, and control the motion characteristics of the prosthesis by changing the gear transmission ratio. Compared with the knee joint of single axis prosthesis, the gear five bar mechanism adopted in document [16] is more complex in structure, but the instantaneous center track of knee prosthesis is closer to that of the human knee joint, and it can effectively realize walking. In terms of design and effect, these multi-bar mechanisms are more suitable for the motion characteristics of human knee joints than single axis prostheses, but they cannot meet the more complex motion.

Based on the analysis of the existing gait acquisition technology, this paper uses Beijing Metric Technology Nokov (metric) optical three-dimensional motion capture system to collect motion data on the lower limbs of the human body, decompose the human motion, analyze and compare the data. By studying the existing prosthetic knee joints, we can conclude that most of the prosthetic knee joints have fixed structural characteristics and an insufficient bionic performance. In the previous work, we have designed a geared five-bar mechanism for prosthetic knee joints [16]. In reference [17] the feasibility of the testing mechanism is analyzed by kinematics and finite element analysis. Through experiments and research, it is found that its performance can meet the corresponding requirements to a certain extent. In this paper, we propose a six-bar mechanism prosthetic knee joint, which uses the motion of the thighs and shanks in the knee joints. ICR (instantaneous center rotation) trajectory of the knee was taken as the optimization target, and the mechanism of the six-bar prosthetic knee joint was designed and optimized, and its bionic performance was experimentally verified.

The rest of this paper is organized as follows: Section 2 uses Nokov (metric) optical three-dimensional motion capture system to collect multi-gait motion data of the lower limbs of the human body; Section 3 expands the mechanism design and optimization of the lower limb prosthesis; in Section 4, the gait test experiments of the prosthesis are illustrated; and in Section 5, conclusions and future works are provided.

## 2. Data Collection and Characteristic Analysis of Human Lower Limb Movement

**Figure 1:**
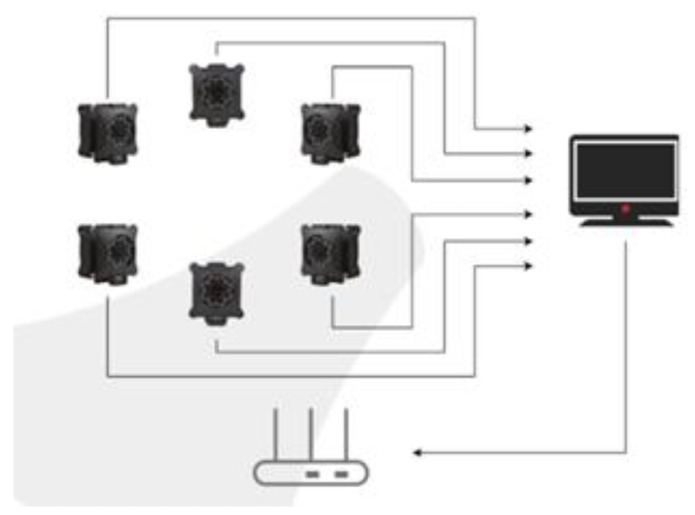
Structure of the Nokov system.

Motion capture technology can quickly record the trajectories of human bodies for real-time or time-delay analysis. The captured information can generate the spatial position of human bodies and objects at a certain moment. Nokov (China’s Beijing Nokov Science & Technology Co.,Ltd.) optical three-dimensional motion capture system is as shown in Figure 1.

**Figure 2:**
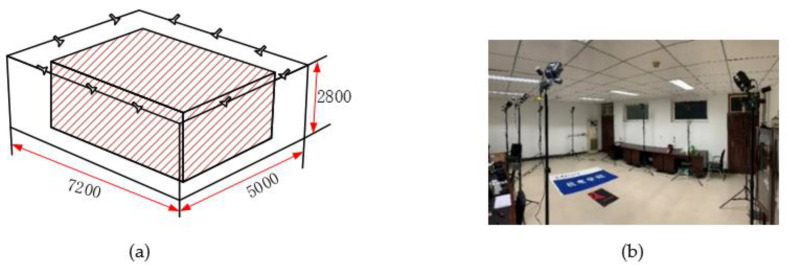
Configuration scheme. (**a**) Layout space (**b**) Experimental setup.

The Nokov optical three-dimensional motion capture system is actually based on computer graphics principles, using several cameras in space to record marked points and then processing them with a computer to obtain data such as the coordinates, velocity, and acceleration of moving objects in space. This system also can provide real-time feedback. According to the recommended layout and the actual situation, we have arranged a layout of 5 m×7.2 m×2.8 m@10Cameras as shown in Figure 2, and the reliable positioning coverage can reach more than 90%. 

**Figure 3:**
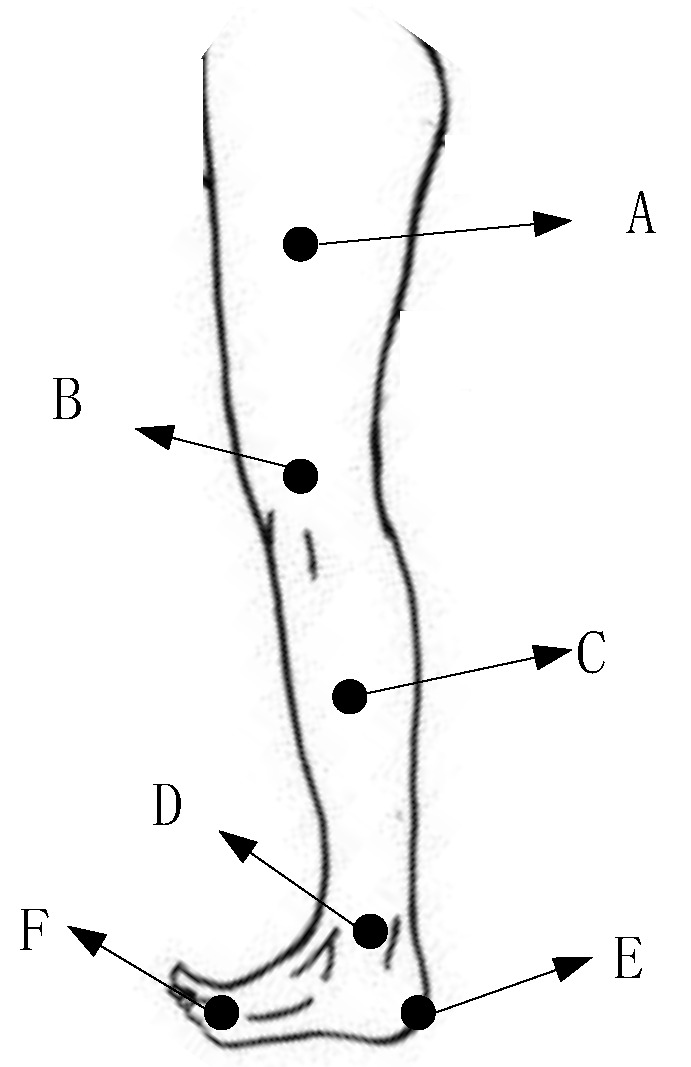
Location of the lower extremity collection mark (A—thigh; B—knee; C—calf; D—ankle; E—heel; F—front foot).

A Nokov motion information collection system was established, and 30 bone healthy and normal gaiters were selected to collect motion data on their knee and ankle joints. This paper mainly studies the motion status of the lower limbs, namely the motion data of the knee joint and the ankle joint, and then marks the positions shown in Figure 3 in combination with the human body structure. 

**Figure 4:**
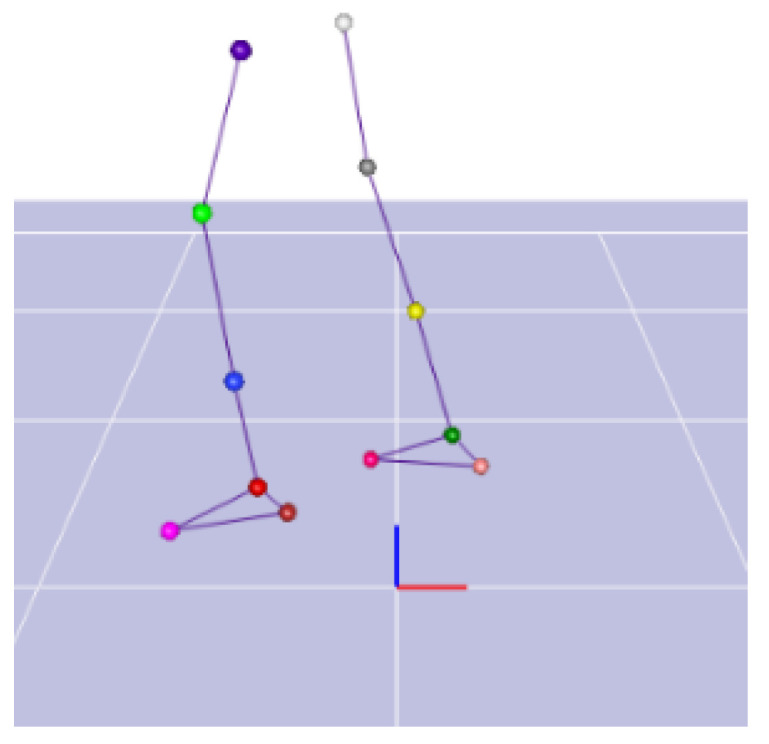
Gait acquisition interface.

The gait acquisition interface is shown in Figure 4.

**Figure 5:**
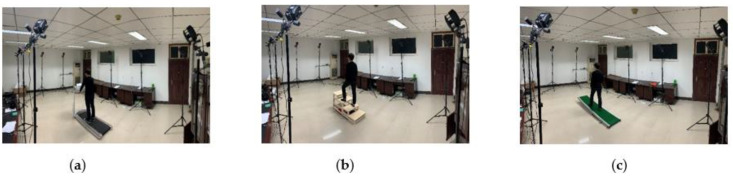
Experiment of motion information collection (**a**) Walking (v=1 m/s) (**b**) Up-stairs (**c**) Up-slope (5°).

Walking, going up and down stairs, and going up and down slopes are the most regular and common actions of people in life. In this paper, a data acquisition is obtained for these gaits. It is assumed that the walking pace of a normal person is 1 m/s, the stairs are a standard four-step model, and the slope is 5°, as shown in Figure 5.

**Figure 6:**
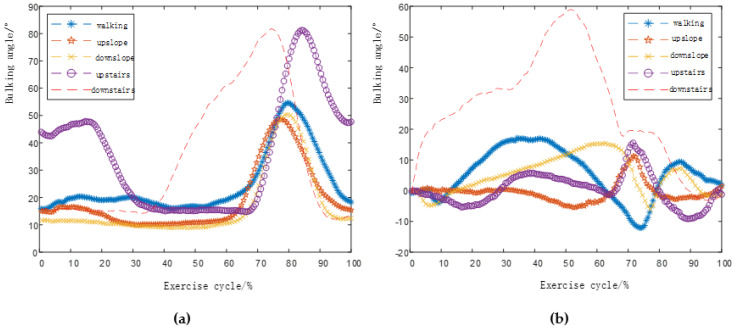
Five gait joint angle changes under time unification. (**a**) Knee joint angle change diagram, (**b**) Ankle joint angle change diagram.

We collected motion data for five gaits of human walking on flat ground *v* = 1 m/s, uphill and downhill (5°), and up and down stairs (4th floor), and unified the time to obtain knee and ankle flexion angles such as Figure 6.

## 3. Mechanism Design and Optimization of Lower Limb Prostheses

### 3.1. Mechanism Selection and Motion Analysis

**Figure 7:**
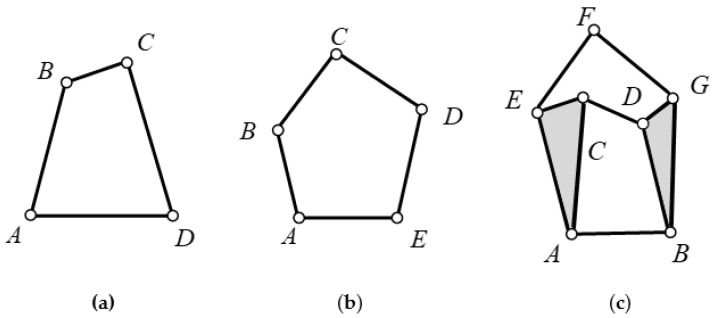
Common mechanism of multi-rod mechanism in prosthesis. (**a**) 4-bar mechanism, (**b**) 5-bar mechanism, (**c**) 6-bar mechanism.

The multi-rod mechanism commonly used in knee joint prostheses is mainly a 4-bar mechanism, a 5-bar mechanism, and a 6-bar mechanism. The 4-bar mechanism shown in Figure 7a is relatively simple, has good reliability, and has a degree of freedom of one. However, the motion curve is relatively single and the bionics are poor. As shown in Figure 7b, although the 5-bar mechanism has a relatively rich motion curve, its degree of freedom is 2, which will make the control of the knee joint more difficult and increase the quality. As shown in Figure 7c, compared with the 4- and 5-bar mechanisms, the 6-bar mechanism not only has a rich motion trajectory, but also has a degree of freedom of 1, which can better simulate the movement of the human knee joint. Therefore, the 6-bar mechanism was selected as the configuration of the knee prosthesis.

The ankle joint is the basic joint of human movement, and the movement is relatively simple. Unlike the human knee joint movement mode, the ankle joint can assist the human lower limbs to complete basic movements through simple uniaxial rotation. Therefore, no complicated multi-rod mechanism is required.

### 3.2. Optimal Design of Knee Joint Prosthesis with Six-Bar Mechanism

#### 3.2.1. Vector Model of Six-Bar Knee Prosthetic Mechanism

**Figure 8:**
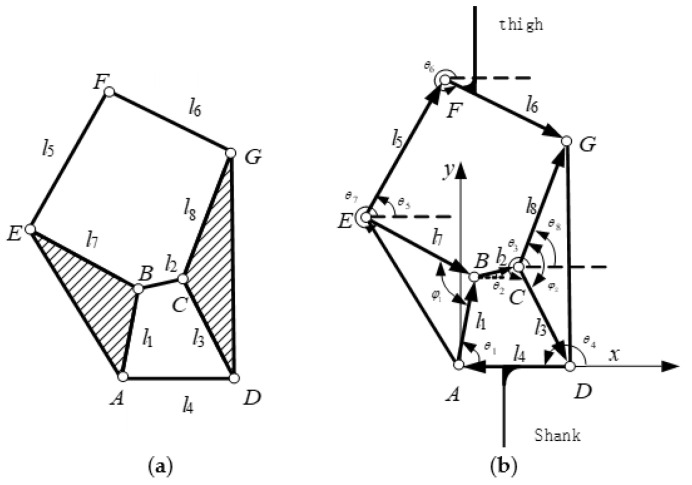
6-bar mechanism model. (**a**) Sketch of 6-bar mechanism (**b**) Vector illustration of 6-bar mechanism.

In fact, the six-bar mechanism model is a Stephenson type I mechanism, which is composed of a four-bar mechanism and a five-bar mechanism with closed motion chains. As shown in Figure 8a:

Based on the schematic diagram of the six-bar mechanism structure, a vector model of the knee prosthesis of the six-bar mechanism is shown in Figure 8b. The rod 4 is fixed on the X axis, and A is the origin of the coordinates to establish a Cartesian coordinate system. The 6-bar mechanism has a total of 10 parameters, which are eight rod lengths $\left(l_{1},l_{2},l_{3},l_{4},l_{5},l_{6},l_{7},l_{8}\right)$ and two triangle initial angles ($\varphi_{1},\varphi_{2}$). Among them, $\left(\theta_{1},\;\theta_{2},\;\theta_{3},\;\theta_{4},\;\theta_{5},\;\theta_{6},\;\theta_{7},\;\theta_{8}\right)$ represent the angle between each vector and the positive direction of the *X* axis. Thigh fix with bar 6, and the shank fix with bar 4. Establish vector equations for closed-chain motions of 4-bar and 5-bar mechanisms:

(1) $$\{\begin{array}{c} {\vec{l}}_{1}+{\vec{l}}_{2}+{\vec{l}}_{3}+{\vec{l}}_{4}=0\;\;\;\;\\ {\vec{l}}_{7}+{\vec{l}}_{2}+{\vec{l}}_{8}-{\vec{l}}_{5}-{\vec{l}}_{6}=0 \end{array}$$

In order to describe the movement position of the six-bar mechanism, a direct description method can be used, and the vector position equation can be established by using the angle relationship between each vector and the positive direction of the X axis to obtain the position of the six-bar mechanism at any buckling angle.

(2) $$\left\{ \begin{array}{l} l_{1}\cos\theta_{1}+l_{2}\cos\theta_{2}+l_{3}\cos\theta_{3}+l_{4}\cos\theta_{4}=0\\ l_{1}\sin\theta_{1}+l_{2}\sin\theta_{2}+l_{3}\sin\theta_{3}+l_{4}\sin\theta_{4}=0\\ l_{7}\cos\theta_{7}+l_{2}\cos\theta_{2}+l_{8}\cos\theta_{8}-l_{5}\cos\theta_{5}-l_{6}\cos\theta_{6}=0\\ l_{7}\sin\theta_{7}+l_{2}\sin\theta_{2}+l_{8}\sin\theta_{8}-l_{5}\sin\theta_{5}-l_{6}\sin\theta_{6}=0 \end{array}\right.$$

In fact, the bars 1 and 7 and bars 3 and 8 in the equation belong to the same component. According to the initial angle provided, we can get:

(3) $$\{\begin{array}{l} -\theta_{7}+\theta_{1}=\varphi_{1}\\ -\theta_{3}+\theta_{8}=\varphi_{2} \end{array}$$

Substituting Equation (3) into Equation (2) gives:

(4) $$\left\{ \begin{array}{l} l_{1}\cos\theta_{1}+l_{2}\cos\theta_{2}+l_{3}\cos\theta_{3}+l_{4}\cos\theta_{4}=0\\ l_{1}\sin\theta_{1}+l_{2}\sin\theta_{2}+l_{3}\sin\theta_{3}+l_{4}\sin\theta_{4}=0\\ l_{7}\cos(\theta_{1}-\varphi_{1})+l_{2}\cos\theta_{2}+l_{8}\cos(\theta_{3}+\varphi_{2})-l_{5}\cos\theta_{5}-l_{6}\cos\theta_{6}=0\\ l_{7}\sin(\theta_{1}-\varphi_{1})+l_{2}\sin\theta_{2}+l_{8}\sin(\theta_{3}+\varphi_{2})-l_{5}\sin\theta_{5}-l_{6}\sin\theta_{6}=0 \end{array}\right.$$

Solving the equations can get the parameter values such as the length and rotation angle of each link.

#### 3.2.2. Analysis of the Instantaneous Center Trajectory of Six-Bar Prosthetic Knee Joint

**Figure 9:**
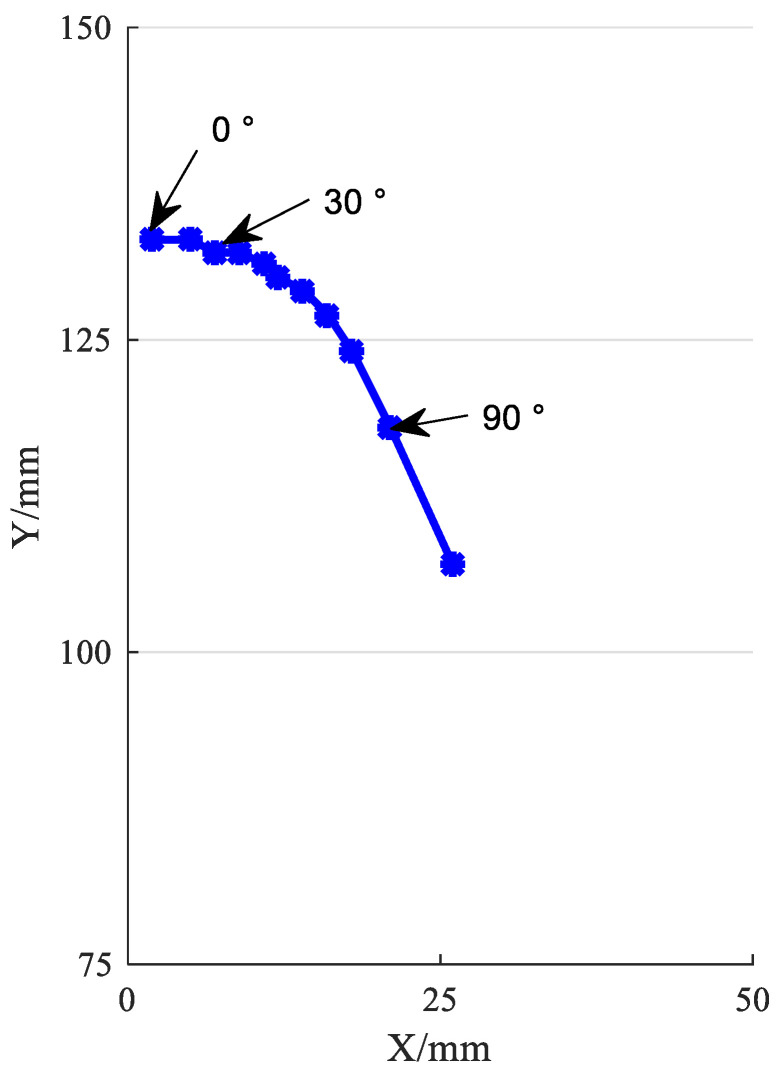
Ideal instant center line of human knee joint (calf fixed).

The center of rotation of a normal human knee joint changes with the change of the gait, which is a composite movement of sliding and rolling, so the instantaneous center curve with a J shape as shown in Figure 9 is formed [18], converted into coordinates (shank fixed) as shown in Table 1.

From Table 1, we can observe a total of 11 sets of data, which represent the instant center position (shank fixed) corresponding to every 10 degrees of knee flexion, and it can be found that when the knee flexion angle increases, the distance of the instant center point will also increase.

**Figure 10:**
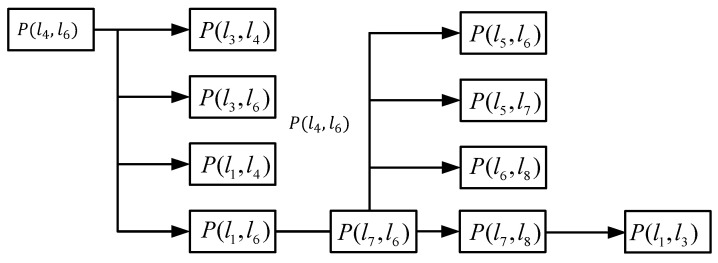
Recursive relationship between instant center points.

We must determine the instantaneous center line of the knee joint prosthesis of the 6-bar mechanism to fit the instantaneous center line of normal human body more accurately. The connecting thigh is rod 6, and the lower leg is rod 4. For the instantaneous center point, the position of the two components that are not directly connected by the motion pair is determined according to the “three-center theorem” (reference). The so-called three-center theorem means that the three instant centers of three components that move in a plane are located on the same straight line. Because only three instant centers are located on the same straight line, it is possible to satisfy the instant centers as isokinetic coincidence points and conditions. We can find the instantaneous point (*X*, *Y*) through the three-center theorem. The reverse logic relationship is shown in Figure 10.

Set point *A* as the origin of the coordinates, and change the coordinates of each point:

(5) $$\left\{ \begin{array}{l} X_{A}=0,Y_{A}=0\\ X_{B}=l_{1}\cos\theta_{1}+X_{A},Y_{B}=l_{1}\sin\theta_{1}+Y_{A}\\ X_{C}=l_{2}\cos\theta_{2}+X_{B},Y_{C}=l_{2}\sin\theta_{2}+Y_{B}\\ X_{D}=-l_{4}\cos\theta_{4},Y_{D}=-l_{4}\sin\theta_{4}\\ X_{E}=l_{7}\cos\theta_{7}+X_{B},Y_{E}=l_{7}\sin\theta_{7}+Y_{B}\\ X_{F}=l_{5}\cos\theta_{5}+X_{E},Y_{F}=l_{5}\sin\theta_{5}+Y_{E}\\ X_{G}=l_{8}\cos\theta_{8}+X_{C},Y_{G}=l_{8}\sin\theta_{8}+Y_{C} \end{array}\right.$$

Find the intersection point *H* of *l_bc_* and *l_ad_* (the instantaneous center):

(6) $$\left[\begin{array}{l} Y_{C}-Y_{B},-\left(X_{C}-X_{B}\right)\\ Y_{D}-Y_{A},-\left(X_{D}-X_{A}\right) \end{array}\right]\left[\begin{array}{l} X_{H}\\ Y_{H} \end{array}\right]=\left[\begin{array}{l} X_{B}\left(Y_{C}-Y_{B}\right)-Y_{B}\left(X_{C}-X_{B}\right)\\ X_{A}\left(Y_{D}-Y_{A}\right)-Y_{A}\left(X_{D}-X_{A}\right) \end{array}\right]$$

Find the intersection point H of *l_hg_* and *l_ef_* (the instantaneous center):

(7) $$\left[\begin{array}{l} Y_{G}-Y_{H},-\left(X_{G}-X_{H}\right)\\ Y_{F}-Y_{E},-\left(X_{F}-X_{E}\right) \end{array}\right]\left[\begin{array}{l} X_{L}\\ Y_{L} \end{array}\right]=\left[\begin{array}{l} X_{H}\left(Y_{G}-Y_{H}\right)-Y_{H}\left(X_{G}-X_{H}\right)\\ X_{E}\left(Y_{F}-Y_{E}\right)-Y_{E}\left(X_{F}-X_{E}\right) \end{array}\right]$$

Solving the equation can obtain the instantaneous center point p46 coordinates (*X*, *Y*):

(8) $$\left[\begin{array}{l} Y_{A}-Y_{L},-\left(X_{A}-X_{L}\right)\\ Y_{G}-Y_{D},-\left(X_{G}-X_{D}\right) \end{array}\right]\left[\begin{array}{l} X\\ Y \end{array}\right]=\left[\begin{array}{l} X_{L}\left(Y_{A}-Y_{L}\right)-Y_{L}\left(X_{A}-X_{L}\right)\\ X_{D}\left(Y_{G}-Y_{D}\right)-Y_{D}\left(X_{G}-X_{D}\right) \end{array}\right]$$

### 3.3. Six-Bar Prosthetic Knee Joint Optimization Results and Motion Analysis

#### 3.3.1. Parameter Optimization

If the instantaneous trajectory of a 6-bar knee prosthesis is similar to the instantaneous trajectory of a human knee, the better the bionic performance is. From this point of view, the least square difference method is used to establish the target of optimization which is the Square of the difference between the instantaneous center point coordinates of the thighs and shank (instantaneous center coordinates of rods 4 and 6 and the ideal instantaneous center coordinates of the human knee):

(9) $$\left(x_{i},y_{i}\right)=F\left(l_{1},l_{2},l_{3},l_{4},l_{5},l_{6},l_{7},l_{8},\varphi_{1},\varphi_{2},\theta_{6}\right)=\min\sum_{i}^{11}\sqrt{{\left(x_{i}-X_{i}\right)}^{2}+{\left(y_{i}-Y_{i}\right)}^{2}}$$

$\left(x_{i},y_{i}\right)$ is the ideal instantaneous center coordinate, and $\left(X_{i},Y_{i}\right)$ is the instantaneous center coordinate of rod 4 and rod 6.

#### 3.3.2. Optimization Method

**Figure 11:**
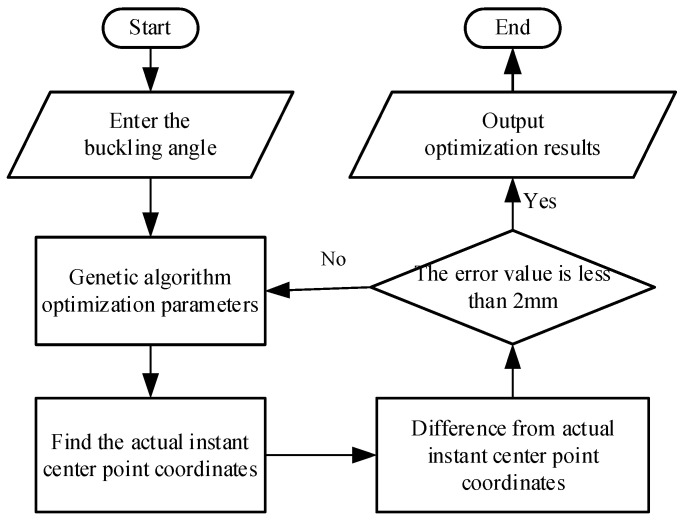
Optimization flowchart.

We use genetic algorithms for optimization. Not only can we get numerical results quickly, but the accuracy will also get higher and higher. The knee joint prosthetic six-bar mechanism has a total of 10 basic parameters $\left(l_{1},l_{2},l_{3},l_{4},l_{5},l_{6},l_{7},l_{8},\theta_{1},\theta_{2}\right)$ and an initial angle $\theta_{6}$. According to the vector equation, the coordinates of the end points of each rod can be obtained, and then the instantaneous center coordinates of the thigh and shank are obtained according to the three-center theorem. The driving angle is selected as the thigh flexion angle, that is, an instant center coordinate is obtained for every 10 degrees of rotation. The optimization process is shown in Figure 11.

It is worth noting that, $\varphi_{1},\varphi_{2}$ as a corner of the triangle, its value range directly affects the final optimization result. When $\varphi_{1},\varphi_{2}$ is less than π, the direction of the triangle will not change. When $\varphi_{1},\varphi_{2}$ is greater than π, the direction of the triangle will change. Therefore, we have classified these two parameters here.

**Figure 12:**
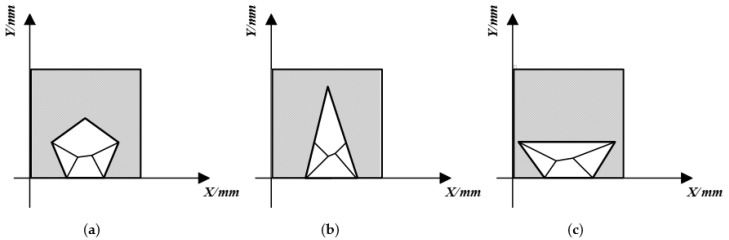
Restricted area of the six-bar mechanism. (**a**) Normal position, (**b**) *Y*-direction limit position, (**c**) *X*-direction limit position.

According to the measurement, the side area of the normal human knee joint is approximately $150\times 150\sim 240\times 240\;{\mathrm{mm}}^{2}$, and the 6-bar mechanism has two extreme positions in the *X* and *Y* directions, as shown in Figure 12b,c. Let the length of the side of the shadow rectangle be *L* and the length of the side of the regular pentagon be *l*. It needs to satisfy $\sqrt{15}/2l<L$ in the *X* direction and 2×l<L in the *Y* direction. Since *L* satisfies 150≤L≤240 mm, the maximum value of 240 mm is taken as the value of L here. Secondly, according to the rationality of the mechanical structure, the minimum length of the pole is given as 20 mm. So the side length needs to meet: $20<l_{i}<120\;\mathrm{mm}\;\left(i=1,2,3,4,5,6,7,8\right)$.

#### 3.3.3. Optimization Results

**Figure 13:**
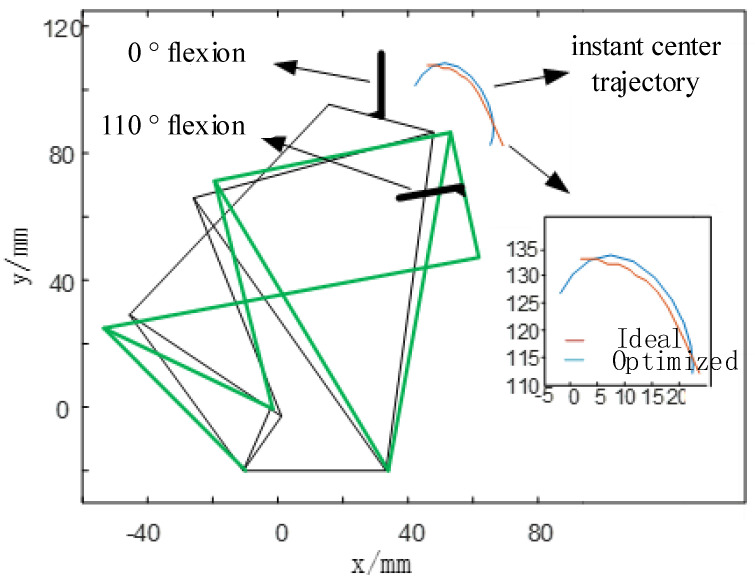
Position of the 6-bar knee prosthesis when it is upright and flexed at 110°.

Because the genetic algorithm optimization results are not necessarily the same each time, through a large number of iterations, we obtain six sets of optimal solutions as shown in Table 2:

In order to further explain the optimization effect, the initial and final states of flexion of the 6-bar knee prosthesis are compared as shown in Figure 13. The figure shows the initial state of the mechanism (0° flexion) and the final state (110° flexion), and the optimized instant center trajectory and ideal instant center trajectory. From the figure, it can also be more intuitively found that the instantaneous center of motion of the thighs and lower legs in the knee joint prosthesis of the optimized 6-bar mechanism is closer to the ideal instant center, indicating that the mechanism has good bionics. And the flexion range of 0° to 110° not only meets the normal walking needs of the lower limb disabled people, but also can achieve large flexion angles such as going up and down stairs and squatting.

## 4. Experimental Research

### 4.1. Experimental Prototype

**Figure 14:**
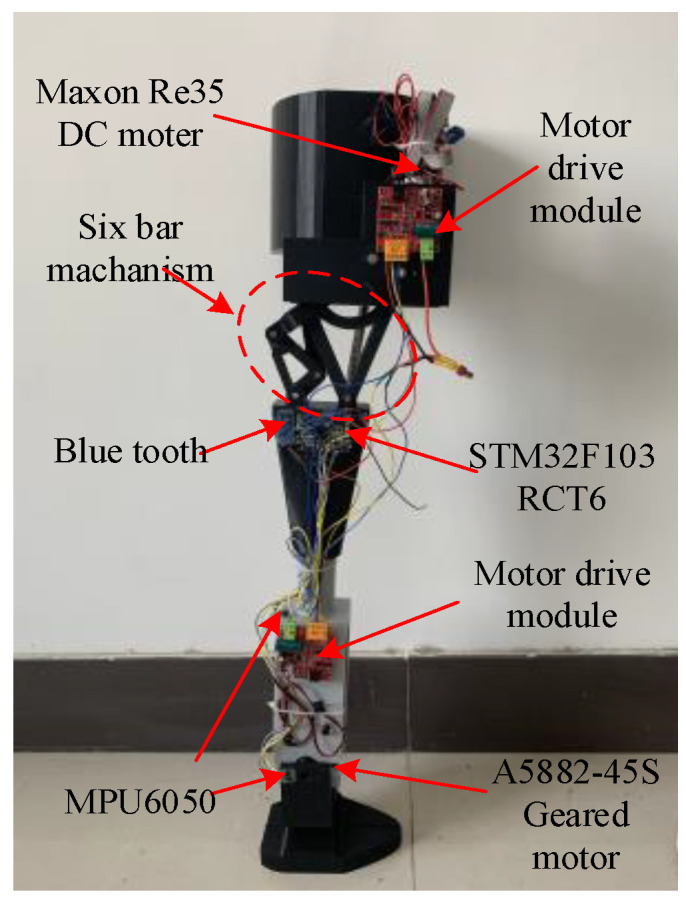
Prosthesis model.

Obtain the structure optimization results of the knee joint, and design the prosthesis experimental prototype, as shown in Figure 14. In order to achieve tracking control of the knee and ankle joint angle in different working modes, first set different tasks in the microcontroller. The modes (walking, up and down stairs, and up and down slopes) correspond to the time-varying expressions of the joint angle. During work, the deviation of the expected joint angle from the actual joint angle is calculated according to the cycle time, and the two motor drive signals required by the PID control pattern are calculated. The DC motor is driven to rotate by two PWM signals, so that the knee and ankle joints can track the expected pattern of motion, and finally the data is transmitted to the PC by the Bluetooth module.

### 4.2. Experimental Process and Result Analysis

**Figure 15:**
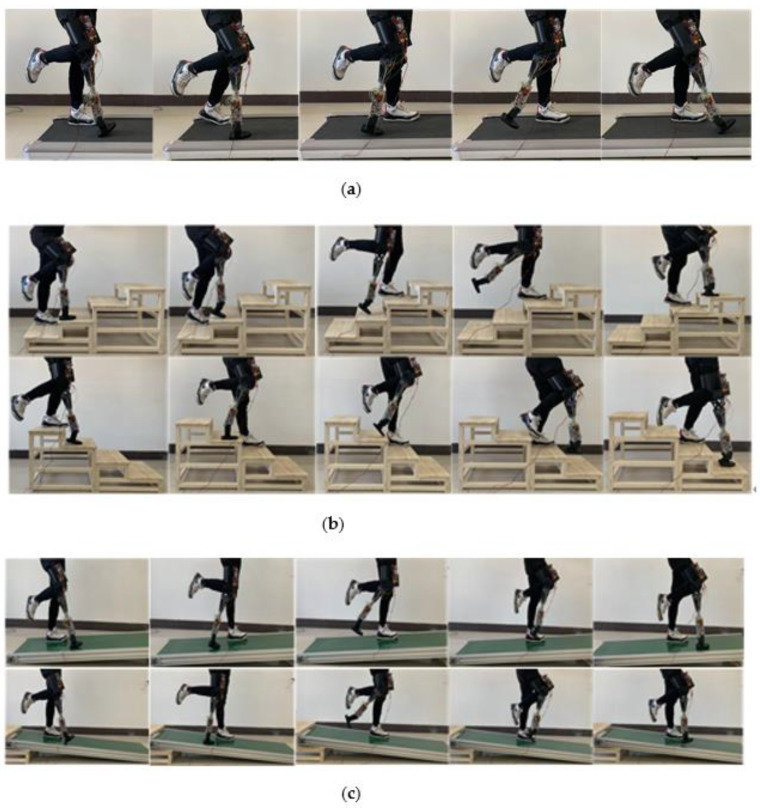
Prosthetic gait experiment. (**a**) Gait experiment of prosthetic flat walking (speed 1 m/s). (**b**) Gait experiment of prosthetic leg up and down steps (four steps). (**c**) Gait experiment of prosthetic ups and downs (gradient 5 degrees).

In this paper, the prosthetic prototyping designed is used to collect motion data for flat walking speed, up and down slopes, and up and down stairs. In order to compare with the data collected in the second part, the experimental conditions are exactly same as in the second part. The experimental process is shown in Figure 15.

**Figure 16:**
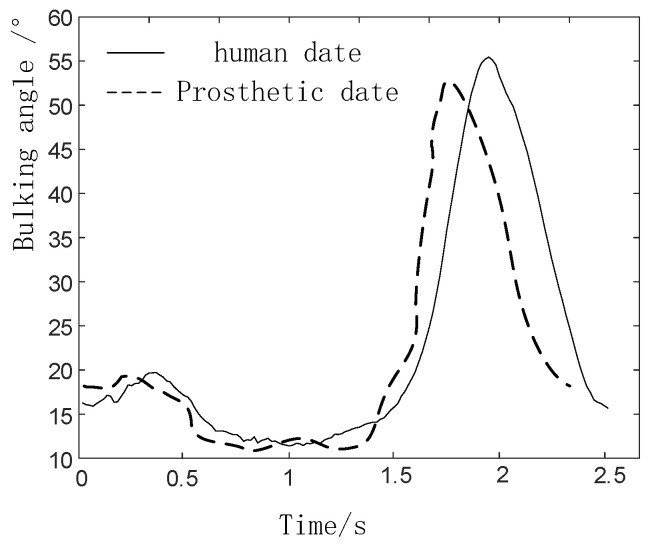
Comparison of knee prosthesis data and human data in walking gait.

**Figure 17:**
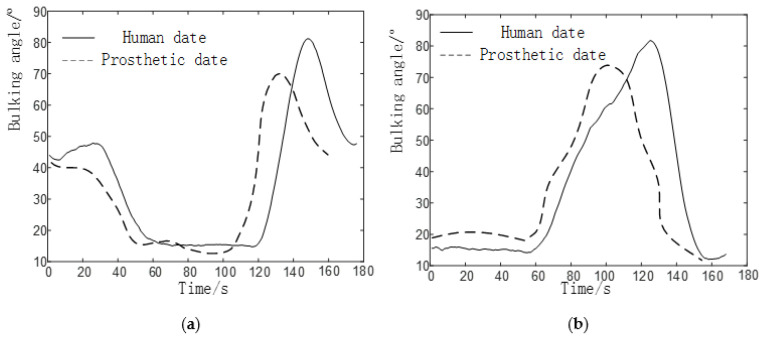
Comparison of knee prosthesis data and human data. (**a**) Knee joint angle change on the upstairs. (**b**) Knee joint angle change on the downstairs.

**Figure 18:**
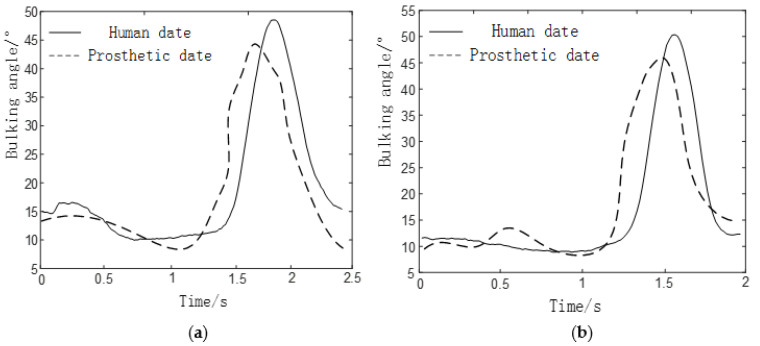
Comparison of knee and ankle prosthesis data and human data. (**a**) Changes in knee angle on uphill knees. (**b**) Changes in knee angle on downhill knees.

Each gait of the prosthesis collected in the experiment is compared with the data of the normal human body measured in the second part, and the results are shown in Figure 16, Figure 17 and Figure 18.

In the process of walking on level ground, the flexion angle trajectory of knee joint prosthesis is roughly the same as that of normal people’s knee joint flexion angle trajectory, but the problem of gait incoordination still exists. The reason for the problem may be that the experimenter was relatively unfamiliar when using the prosthesis for the first time and failed to adapt well to the prosthesis, so some errors will inevitably be caused during the experiment.

In the process of going up and down the stairs, the flexion angle trajectory of the knee joint prosthesis is similar to that of the normal knee flexion angle trajectory, but the amplitude is small, the trajectory is delayed, and the standing phase period is shortened. The reasons for the problem are:

1. The joint of a normal person provides a large driving torque(about 110Nm) in the standing phase. However, due to the insufficient strength of the component materials in this experiment, the joint prosthesis failed to provide a large driving torque in the standing phase.
2. Due to the error between the actual weight of the prosthesis and the theoretical weight, the joint drive is insufficient, which has a certain negative impact on the result.

In the process of up and downhill, the flexion angle trajectory of the knee joint prosthesis is closer to the overall trend of the normal people’s knee flexion angle trajectory, but there are also problems such as small amplitude, and the flexion angle of knee joint and ankle joint is lower than that of normal people (50°). The cause of the problem is roughly the same as that of the prosthetic gait going up and down stairs.

From the above results, the analysis shows that the walking gait of the knee joint prosthesis is composed of 6-bar mechanism, the knee joint angle change curve of up and down stairs and up and down slopes are similar to the normal people’s knee joint angle curves, which can meet the basic requirements. The small errors appear mainly due to the following reasons:

1. The experimenter did not adapt well to the prosthesis and caused gait inconsistency.
2. The error between the actual weight of the prosthesis and the theoretical weight.

## 5. Discussion

In this paper, 30 young subjects were selected, and the motion data of knee and ankle joints were collected by a Nokov(metric) optical three-dimensional motion capture system, and the motion data of knee and ankle joints were obtained under multiple gaits. After optimizing the structure of the proposed six-bar prosthetic knee joint model by using genetic algorithm, the experimental prototype was designed to carry out relevant experimental tests. Through the experiment, it is found that the flexion angle trajectory of the prosthesis proposed in this paper has the same general trend as the flexion angle trajectory of the normal knee and ankle joint, which is basically at about 55°. When going up and down stairs and downhill, the buckling angle trajectories of the two models are basically close to each other, but the amplitude of the buckling angle trajectories of the six-bar prosthetic knee joint model is lower than the theoretical value (50°), which is basically about 45°. Although this study has some limitations, the prosthetic knee can help patients achieve a variety of gaits and smooth movement, which can be improved by improving the component materials and weight of the prosthetic limb, and has great potential in other directions, such as prosthetic and robotic design.

## Figures and Tables

**Table 1:** Ideal instant center point coordinates.

Buckling Angle (°)	Instantaneous Center Coordinates (mm)	Buckling Angle (°)	Instantaneous Center Coordinates (mm)
0	(2133)	10	(5133)
20	(7132)	30	(9132)
40	(11,131)	50	(12,130)
60	(14,129)	70	(16,127)
80	(18,124)	90	(21,118)
100	(26,107)	110	(31,87)

**Table 2:** Optimization results.

Parameters	$l_{1}$	$l_{2}$	$l_{3}$	$l_{4}$	$l_{5}$	$l_{6}$	$l_{7}$	$l_{8}$	$\varphi_{1}$	$\varphi_{2}$	$\theta_{6}$
length	28.7	76.9	96.7	51.4	116.6	27.8	63.5	90.8	17.69	−1.4	24.7

## Data Availability

Not Applicable.
